# Low-density polyethylene microplastics alter chemical properties and microbial communities in agricultural soil

**DOI:** 10.1038/s41598-023-42285-w

**Published:** 2023-09-28

**Authors:** Kumuduni Niroshika Palansooriya, Mee Kyung Sang, Ali El-Naggar, Liang Shi, Scott X. Chang, Jwakyung Sung, Wei Zhang, Yong Sik Ok

**Affiliations:** 1 Korea Biochar Research Center, APRU Sustainable Waste Management Program & Division of Environmental Science and Ecological Engineering, Korea University, Seoul, 02841 Republic of Korea; 2 Department of Renewable Resources, University of Alberta, Edmonton, AB T6G 2E3 Canada; 3 State Key Laboratory of Subtropical Silviculture, Zhejiang A&F University, Hangzhou, 311300 China; 4 Division of Agricultural Microbiology, Rural Development Administration, National Institute of Agricultural Science, Wanju, 55365 Republic of Korea; 5 Department of Soil Sciences, Faculty of Agriculture, Ain Shams University, Cairo, 11241 Egypt; 6 College of Life Sciences, Nanjing Agricultural University, Nanjing, 210095 China; 7 Department of Crop Science, College of Agriculture, Life Science and Environmental Chemistry, Chungbuk National University, Cheongju, 28644 Chungcheongbuk-Do Republic of Korea; 8 Institute of Environmental Research at Greater Bay Area, Key Laboratory for Water Quality and Conservation of the Pearl River Delta, Ministry of Education, Guangzhou University, Guangzhou, 510006 China; 9 Institute of Green Manufacturing Technology, College of Engineering, Korea University, Seoul, 02841 Republic of Korea

**Keywords:** Biogeochemistry, Environmental sciences

## Abstract

Microplastic (MP) pollution in agricultural soils, resulting from the use of plastic mulch, compost, and sewage sludge, jeopardizes the soil microbial populations. However, the effects of MPs on soil chemical properties and microbial communities remain largely unknown. Here, we investigated the effects of different concentration levels (0, 0.1, 1, 3, 5, and 7%; w:w) of low-density polyethylene (LDPE) MPs on the chemical properties and bacterial communities of agricultural soil in an incubation study. The addition of LDPE MPs did not drastically change soil pH (ranging from 8.22 to 8.42). Electrical conductivity increased significantly when the LDPE MP concentrations were between 1 and 7%, whereas the total exchangeable cations (Na^+^, K^+^, Mg^2+^, and Ca^2+^) decreased significantly at higher LDPE MP concentrations (3–7%). The highest available phosphorus content (2.13 mg kg^−1^) was observed in 0.1% LDPE MP. Bacterial richness (Chao1 and Ace indices) was the lowest at 0.1% LDPE MP, and diversity indices (Shannon and Invsimpson) were higher at 0 and 1% LDPE MP than at other concentrations. The effect of LDPE MP concentrations on bacterial phyla remained unchanged, but the bacterial abundance varied. The relative abundance of Proteobacteria (25.8–33.0%) was the highest in all treatments. The abundance of Acidobacteria (15.8–17.2%) was also high, particularly in the 0, 0.1, and 1% LDPE MPs. With the increase in LDPE MP concentration, the abundance of Actinobacteria gradually increased from 7.80 to 31.8%. Our findings suggest that different MP concentration levels considerably alter soil chemical properties and microbial composition, which may potentially change the ecological functions of soil ecosystems.

## Introduction

Global plastic production has surged from 1.5 million metric tons (Mt) to 367 million Mt over the last 70 years (1950–2020)^1^. The increased use of plastic mulch has resulted in the accumulation of plastic materials in agricultural lands^2, 3^. In China, nearly 20 million hectares of agricultural land are covered with plastic mulch films, and the quantity of plastic mulch reached 1.25 million tons in 2011^4^. Nearly 79% of global plastic waste is piled in landfills^5^, and soil serves as a large sink for microplastics (MPs)^6, 7^. Moreover, the application of sewage sludge as a fertilizer can introduce MPs into agricultural lands^8^. It has been estimated that 63,000–430,000 tons and 44,000–300,000 tons of MPs can enter the soil annually in European and North American agricultural lands, respectively^9^. In addition, the weathering of plastics accumulated in the soil can generate MPs and nanoplastics, which may be harmful to various ecosystems^10, 11^. MP (≤ 5 mm fragments) contamination was listed among the top 10 environmental problems by the United Nations Environment Program in 2014^6^. MPs can negatively affect various soil properties because of their persistent characteristics that pose a threat to important terrestrial ecosystems^12, 13^. However, the pollution risks and environmental impacts of MPs on agricultural soils have not been well documented, although they have received increasing global attention^14–17^.

Polyethylene (PE) is a polymer widely used to produce mulch films and other plastic products used in agriculture^18^. Low-density polyethylene (LDPE), a synthetic resin manufactured by polymerizing ethylene, is used in agriculture, such as in greenhouses and for mulching^19^. LDPE is commonly used because of its versatility, processability, low cost, and flexibility^20^. All these advantages make it an ideal raw material to achieve benefits such as maintaining soil temperature and moisture content and preventing weed growth^21, 22^, all of which ultimately contribute to enhanced agricultural production. However, the widespread use of non-biodegradable LDPE has resulted in serious environmental concerns^22^. Recent research has shown that the presence of LDPE MPs in the soil can alter microbial community characteristics and enzymatic activity^23–25^. Moreover, LDPE has been recognized as a substrate for distinct microbial colonization, which may modify the microbial community structure and hinder ecosystem functioning^26, 27^. These changes can alter the fertility of the soil^28^. Certain bacteria, such as *Arcobacter* and *Colwellia* spp., can colonize LDPE, resulting in the successional formation of plastisphere-specific bacterial assemblages^29^. Nevertheless, the quantity of MPs in the soil is an important factor in determining the soil's chemical properties and microbial activity. Judy et al.^30^ found no remarkable shifts in the soil microbial community and diversity with 1% (w:w) PE, polyvinyl chloride, or polyethylene terephthalate MPs compared to the control (without MPs). In contrast, 5% (w:w) polyvinyl chloride MP considerably changed the abundance of bacterial groups^31^. However, these findings vary among different studies. A few studies have focused on the effects of LDPE MPs across a wide range of concentration levels on the chemical properties and microbial communities in soil. Moreover, correlations among LDPE MP concentrations, soil chemical properties, and microbial communities have rarely been analyzed. Thus, knowledge of the relationships between these properties and their underlying mechanisms remains incomplete, impeding our capacity to address the issues associated with MP pollution in agroecosystems. We hypothesized that LDPE MPs may alter soil chemical properties and microbial community structure and that these changes could differ with varying concentrations and exposure time of LDPE MPs. Thus, the aim of this study was to explore the effect of LDPE MPs across a range of concentration levels on changes in (i) soil chemical properties; (ii) soil bacterial richness, diversity, and abundance; and (iii) correlations among soil chemical properties, bacterial communities, and LDPE MP concentrations using an incubation study. The results of this study enhance our understanding of the potential risks posed by LDPE MPs in agroecosystems. In addition, our findings will be useful for policymakers to develop policies and regulations to minimize plastic-associated environmental issues and to protect soil health.

## Materials and methods

### Soil collection and handling

The soils used for the incubation study were collected from an agricultural field in Busan (35°14ʹ24.5ʺN, 128°58ʹ44.4ʺE), South Korea. The region has a humid subtropical climate with a mean annual temperature of 15.5 °C and a mean annual precipitation of 98 mm. The field has been used to grow soybeans for several years. The soil samples were collected before soybean planting in May 2020. Considering the soil processes, nutrient cycling, microbial activity, and plant-root interactions occurring in the rooting zone, 30 soil samples were randomly collected from a depth of 0–40 cm (representing the rooting zone).

After removing the litter layer from the soil surface, subsamples were collected in a steel container, thoroughly mixed to obtain a composite sample, and then brought to the laboratory. They were spread on ink-free paper for air-drying, which was performed in a dust-free, well-ventilated room at 24 ± 1 °C. Thereafter, plant residues and other organic debris were carefully removed using forceps. The soil was ground and sieved using a 2 mm metallic sieve, homogenized by mixing in a steel container and stored in high-density polyethylene storage containers until chemical analyses and incubation experiments were conducted.

### Preparation of LDPE MPs

The LDPE plastic mulch films, produced by IHLSHIN Chemical (Republic of Korea) were fragmented into pieces (16–2100 µm in size) through a cryomilling process. Briefly, the of LDPE mulch film pieces were embrittled with liquid nitrogen and crushed in an ultracentrifugal mill utilizing a 5 mm ring sieve (Freezer/Mill^®^ Cryogenic grinder 6875). The resulting LDPE MPs were air-dried and stored in Pyrex bottles at 24 ± 1 °C until their use in subsequent experiments.

### Experiment setup

Considering the accumulation of MPs in soil, six LDPE MP concentration levels were selected: 0, 0.1, 1, 3, 5, and 7% (w:w). According to previous research, these MP levels can be considered environmentally relevant under significant human activities, where the MP level can reach up to 7%. A MP addition of < 1% can be classified as a low concentration, while an addition > 1% can be considered a high concentration^32, 33^. The incubation experiment was conducted in an automatic incubator at the laboratory scale. The LDPE MPs were thoroughly mixed with 25 g of soil at the above concentrations and placed in enclosed 50 mL sterilized Falcon tubes (perforated lids were used to allow air exchange). The soil moisture content of each tube was maintained at 70% of the soil water-holding capacity by adding deionized water (based on the weight loss) throughout the experiment. The control samples were filled with soil only (i.e., 0% treatment without adding LDPE MPs). All treatments and control groups were quadruplicated and incubated in the dark at 25 °C for 100 d in an incubator (MIR-554, SANYO Electronic, Co., Ltd., Japan). Following the 100 d incubation period, soil samples were gathered and divided into two subsample groups for chemical and microbial analyses.

### Soil physicochemical properties

The soil pH and electrical conductivity (EC) were determined by dissolving soil samples (collected before and after incubation) in deionized water in a ratio of 1:5 (solid:solution)^34^. Available phosphorus (P) content was measured by the ascorbic acid method^35^ using a UV–VIS spectrophotometer (Thermo Fisher Scientific, USA). Exchangeable cations, including Na^+^, K^+^, Mg^2+^, and Ca^2+^ were extracted using 1 M ammonium acetate (NH_4_CH_3_CO_2_, pH 7) and their concentrations were measured using inductively coupled plasma optical emission spectroscopy (ICP-OES, Optima 7300 DV, Perkin-Elmer, USA)^36^. The sum of Na^+^, K^+^, Mg^2+^, and Ca^2+^ is reported as the total exchangeable cation content. The percentages of sand, silt, and clay were determined using the hydrometer method^37^, and soil texture was determined according to the USDA textural triangle^38^. The physicochemical properties of the soil are available in Dissanayake et al.^13^.

### Soil bacterial community composition

For microbial analysis, soil samples were collected twice: (i) on the first day of incubation (0 d) and (ii) at the end of incubation (100 d). Genomic DNA was extracted from the soil (0.5 g) using the FastDNA^®^ spin kit (MP Biomedicals, USA), according to the manufacturer’s instructions^39^. The concentration and purity of the genomic DNA were measured using a NanoDrop 2000 spectrophotometer (Thermo Fisher Scientific, USA), and the soil DNA was pyrosequenced at Macrogen Inc. (Seoul, Republic of Korea). Briefly, the bacterial 16S gene in the extracted DNA was amplified using universal primers 341F and 805R (341F:5′-CCTACGGGNGGCWGCAG-3′ and 805R:5′-GACTACHVGGGTATCTAATCC-3′). Amplification was performed using the following protocol: initial denaturation at 95 °C for 3 min; 25 cycles of denaturation at 95 °C for 30 s, annealing at 55 °C for 30 s, and extension at 72 °C for 30 s; and final extension at 72 °C for 5 min. The products were normalized and pooled using PicoGreen, and the size of the libraries was verified using the TapeStation DNA Screentape D1000 (Agilent) and sequenced using the MiSeq™ platform (Illumina, San Diego, CA, USA). Based on operational taxonomic units, richness and diversity indices were calculated using MOTHUR^40^, and then statistically separated using the Statistical Analysis System ver. 9.4 (SAS, Cary, NC, USA).

### Quality control and statistical analysis

For quality control and quality assurance (QA/QC) in our research, all glassware was properly cleaned, washed with diluted hydrochloric acid, and rinsed with deionized water before use. The laboratory equipment and work surface were thoroughly cleaned with 70% ethanol and wiped with paper wipes before performing the analysis. Cotton lab coats were worn to prevent MP contamination, and items made of plastic (e.g., plasticware, suits, and fabrics) were avoided during this study. All the analytical instruments were calibrated prior to chemical analysis. Analyses were performed using four experimental replicates, and the results are reported as the mean ± standard deviation. Blank samples and analytical-grade reagents were employed for all the analyses. The values of the blank samples were either low or below the detection limits of the corresponding method. The normal distribution of the data was tested using the PROC UNIVARIATE in SAS ver. 9.4 (Cary, NC, USA). To compare the two groups (0 d vs. 100 d), PROC GLM was performed using SAS ver. 9.4. The significance of differences between treatments was evaluated using one-way analysis of variance (ANOVA). The least significant difference (LSD) test verified significant differences between the various treatments at *p* < 0.05. Principal component analysis (PCA) was performed using SPSS 19.0, to correlate the LDPE MP concentrations with soil chemical properties and bacterial community data.

## Results and discussion

### Effects of LDPE MPs on soil chemical properties

**Figure 1:**
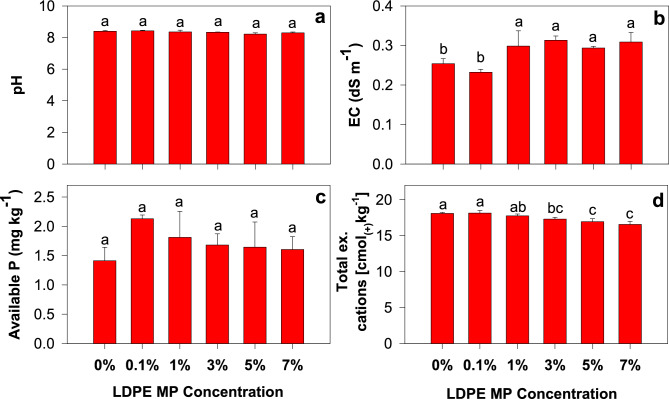
Changes in soil (**a**) pH, (**b**) electrical conductivity (EC), (**c**) available P content, and (**d**) total exchangeable cation content with varied low-density polythylene (LDPE) microplastic (MP) concentrations at 0, 0.1, 1, 3, 5, and 7%. According to the least significant difference (LSD) test, the same letters on bars indicate that pH/EC/available P/total exchangeable cation content are not significantly different at *p* < 0.05.

The soil texture was silty loam, and the initial soil pH was moderately alkaline (8.25). The higher pH at the sampling sites may have been due to the long-term cultivation of soybeans. Liu et al.^41^ reported that soil pH and nutrient availability can be improved after long-term continuous soybean cropping. The pH of soils treated with LDPE MPs ranged from 8.22 to 8.42, with no significant difference among treatments (Fig. 1a), indicating that LDPE MPs (0.1–7%) did not affect the soil pH. This is consistent with the results of Qi et al.^42^, who reported that a short incubation period might not be sufficient for MPs to initiate chemical changes in the soil. The soil EC increased (*p* < 0.05) at higher LDPE MP concentrations (1–7%) (Fig. 1b). In general, if soil EC exceeds 2 dS m^−1^, soil salinity development, crop growth, and performance may be restricted^43^. However, none of the treatments increased soil EC causing soil salinity. The increasing EC values were probably due to enhanced microbial activity at higher LDPE MP levels. Soil microbial communities can enhance the mineralization of soil organic and inorganic compounds, thereby releasing inorganic nutrients that can increase the soil EC^44^. In contrast, Qi et al.^42^, demonstrated that the addition of LDPE plastic debris did not affect soil EC and productivity owing to the short incubation period (1 month).

The LDPE MP treatments increased the available P content in the soil, although no significant differences were found among the treatments (Fig. 1c). The highest value was observed for the 0.1% LDPE MP treatment, with a 50.8% increase compared with that of the control. This was probably due to the changes in soil nutrient content at some LDPE MP concentrations. For example, Liu et al.^3^ reported that MP incorporation into the soil can facilitate the release of soil nutrients such as carbon, nitrogen, and phosphorus into the soil solution. In contrast, the addition of 0.2% PE MPs did not alter the availability of nutrients in the soil, including phosphorus, indicating that environmentally relevant concentrations of MPs did not have an impact on soil nutrient supply^45^. In addition, no significant changes in inorganic P concentrations were observed in soils amended with MPs^46^. Generally, an increase in soil pH can facilitate the precipitation of P with Ca^2+^ (i.e., more crystalline Ca-P), which decreases the amount of soluble phosphate^47^. However, in this study, no significant difference was found in pH; thus, there was no reduction in available P.

Higher LDPE MP concentrations (3, 5, and 7%) resulted in relatively lower total exchangeable cation content than the control and lower LDPE MP concentrations (0.1 and 1%) (Fig. 1d). These results indicate that an increase in MP concentration could decrease essential plant nutrients such as Na^+^, K^+^, Mg^2+^, and Ca^2+^ in the soil. Overall, the concentrations of LDPE MP had no effect on pH and available P, whereas soil EC increased, and total soil exchangeable cation content decreased at higher LDPE MP concentrations.

### Effects of LDPE MPs on soil bacterial richness and diversity

**Figure 2:**
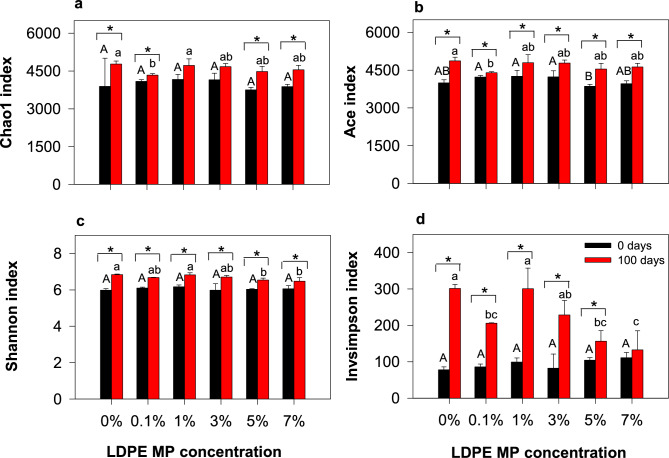
Changes in (**a**) Chao1, (**b**) Ace, (**c**) Shannon, and (**d**) Invsimpson indices before (0 d) and after (100 d) incubation with varied LDPE MP concentrations at 0, 0.1, 1, 3, 5, and 7%. Letters “AB” and “abc” above the bars represent the significant difference among the LDPE treatments. According to LSD, the same letters on two bars indicate that Chao1/Ace/Shannon/Invsimpson index is not significantly different at *p* < 0.05. Asterisk (*) represents the significant difference of *p* < 0.05 between 0 and 100 d.

The Chao1 and Ace indices were used to estimate bacterial richness, and the Shannon and Invsimpson indices were used to evaluate the bacterial diversity of the samples^48^. The Chao1, Ace, Shannon, and Invsimpson indices were significantly increased after 100 d of incubation compared to the initial samples (0 d), except for a few cases (Fig. 2). The increase in soil bacterial richness and diversity after incubation was likely due to changes in soil microbial habitat over time. For instance, prolonged incubation with adequate moisture content (70% of water-holding capacity) in the soil might have increased soil bacterial growth and activity^49^. The availability of soil nutrients (e.g., available P) and carbon supplementation over time can promote microbial activity in the soil, thus enhancing their richness and diversity^50^. Moreover, LDPE can act as a factitious surface for microbial colonization^51^. Therefore, in the present study, different amounts of LDPE MPs incorporated into the soil were expected to improve microbial richness and diversity after 100 d of incubation.

At the end of the incubation period, different concentrations of LDPE MP did not significantly affect the Chao1 and Ace indices, except for the 0.1% LDPE MP treatment (Fig. 2 and Table S1). The addition of 0.1% MP significantly reduced the Chao1 and Ace indices compared with the control, indicating that certain MP amounts can negatively affect bacterial richness in the soil^23, 52^. Shannon and Simpson's indices were relatively lower at 5% and 7% LDPE MP concentrations compared with the control and other treatments. This implies that high concentrations of LDPE MPs can negatively affect some soil bacteria, thereby reducing their diversity in the soil. This may be due to variations in growth factors among different bacterial species^53^ and changes in microbial diversity induced by MPs^15, 54^. Moreover, the presence of high amounts of MPs can exert selection pressure on soil microbes, which can alter the microbial community structure and diversity, while exerting evolutionary consequences^55^. The alterations caused by MPs in soil ecosystems may also lead to the loss of microhabitats for indigenous microorganisms^56^.

### Effects of LDPE MPs on soil bacterial community structure

**Figure 3:**
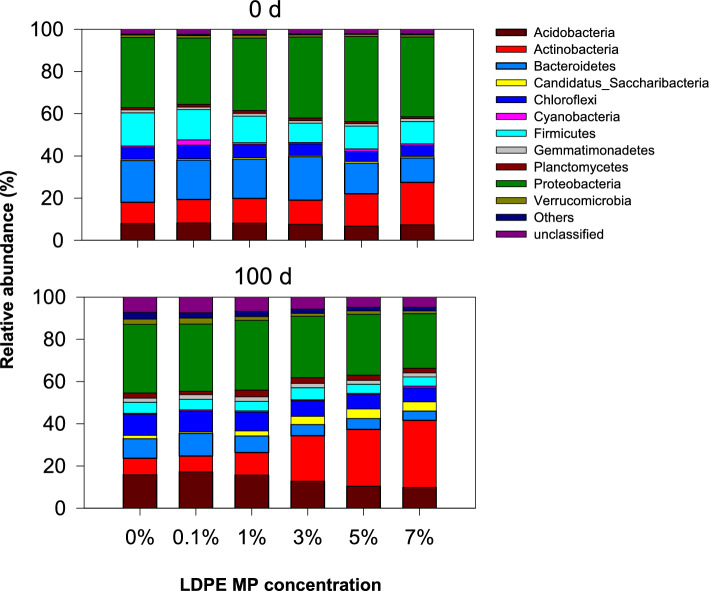
Relative abundance of the bacterial community in the soil before incubation (0 d) and after incubation (100 d) with varied LDPE MP concentrations at 0, 0.1, 1, 3, 5, and 7%.

Eleven phyla (Acidobacteria, Actinobacteria, Bacteroidetes, Candidatus Saccharibacteria, Chloroflexi, Cyanobacteria, Firmicutes, Gemmatimonadetes, Planctomycetes, Proteobacteria, and Verrucomicrobia) as well as a few unspecified communities were identified (Fig. 3 and Table S2). Such bacterial communities were present in both soils (before and after incubation), with changes in their relative abundances (%) depending on the LDPE MP concentration and incubation period. Before incubation (0 d), Proteobacteria (31.4–40.2%) displayed the highest relative abundance and was considered one of the dominant bacterial phyla, followed by Bacteroidetes (11.5–20.6%), Actinobacteria (9.94–20.0%), and Firmicutes (9.11–15.7%) in the control and all LDPE-MP-treated soils. These phyla constitute the dominant strains found globally in soil^57^. The phylum Proteobacteria has substantially high metabolic, physiological, and morphological diversity and can survive under various environmental conditions^58–60^. Proteobacteria are typically observed in soil libraries^61^. Proteobacteria and Actinobacteria are also the most abundant in soils worldwide^62^, and can survive under various extreme environmental conditions, such as cold stress, drought, and heavy metal contamination^63, 64^. Proteobacteria, Acidobacteria, and Actinobacteria are the most common and abundant bacterial phyla in a wide range of forest soils^61^.

After incubation for 100 d, Proteobacteria (25.8–33.0%) displayed the highest relative abundance among the control and all LDPE MP-treated soils. Proteobacteria preferably colonize low bulk density and nutrient-rich soils with high carbon availability^65^. However, the abundance of Proteobacteria decreased in soils treated with higher concentrations of LDPE MPs. Actinobacteria (7.5–31.8%), Acidobacteria (9.8–17.2%), Chloroflexi (6.5–10.0%), and Bacteroidetes (4.3–10.7%) were the most abundant phyla in all treatments. However, Acidobacteria, Bacteroidetes, Chloroflexi, Proteobacteria, and Verrucomicrobia showed greater relative abundances at lower LDPE MP concentrations (< 1%), whereas Firmicutes and Gemmatimonadetes were more abundant at < 3% LDPE MP concentrations. Acidobacteria can survive in MP-contaminated soils subjected to intensive plastic mulching for more than 30 years^26^. Acidobacteria, Bacteroidetes, Gemmatimondadetes, Proteobacteria, and Nitrospirae were considerably increased in soils amended with MPs^23^. Moreover, Firmicutes, Proteobacteria, and Actinobacteria grew in the LDPE MP-contaminated soils. For instance, MPs and As-contaminated soils showed the highest abundance of Proteobacteria^66^. Additionally, PE MP-treated soils showed the highest abundance of Proteobacteria, followed by Firmicutes and Actinobacteria^67^. Moreover, Actinobacteria and Proteobacteria can reportedly degrade plastic mulch films in agricultural lands and are therefore termed plastic-associated bacteria^26^. Interestingly, the relative abundance of Actinobacteria increased as the concentration of the LDPE MPs increased from 0 to 7%. Candidatus Saccharibacteria showed the highest relative abundance (3.9–4.5%) within the 3–5% LDPE MP concentration, whereas Cyanobacteria exhibited the greatest abundance (0.94%) at the 7% LDPE MP concentration. These results imply that different concentrations of LDPE MPs distinctly affect the bacterial species in the soil. Actinobacteria can degrade LDPE, which generates long-chain alkanes as byproducts of bacterial LDPE decay^68^. Several studies have shown that *Streptomyces* (e.g., *Streptomyces fulvissimus*), which is the largest genus of Actinobacteria, can degrade PE in soil^68^. Thus, the increase in substrate-specific bacteria on LDPE MPs shows that LDPE MPs can act as substances for the proliferation of potential plastic-degrading bacteria in the soil. Further studies are essential to explore these reasons, gather additional evidence for the differences in bacterial phyla, and identify the ability of MP-associated bacteria to degrade LDPE MPs.

**Figure 4:**
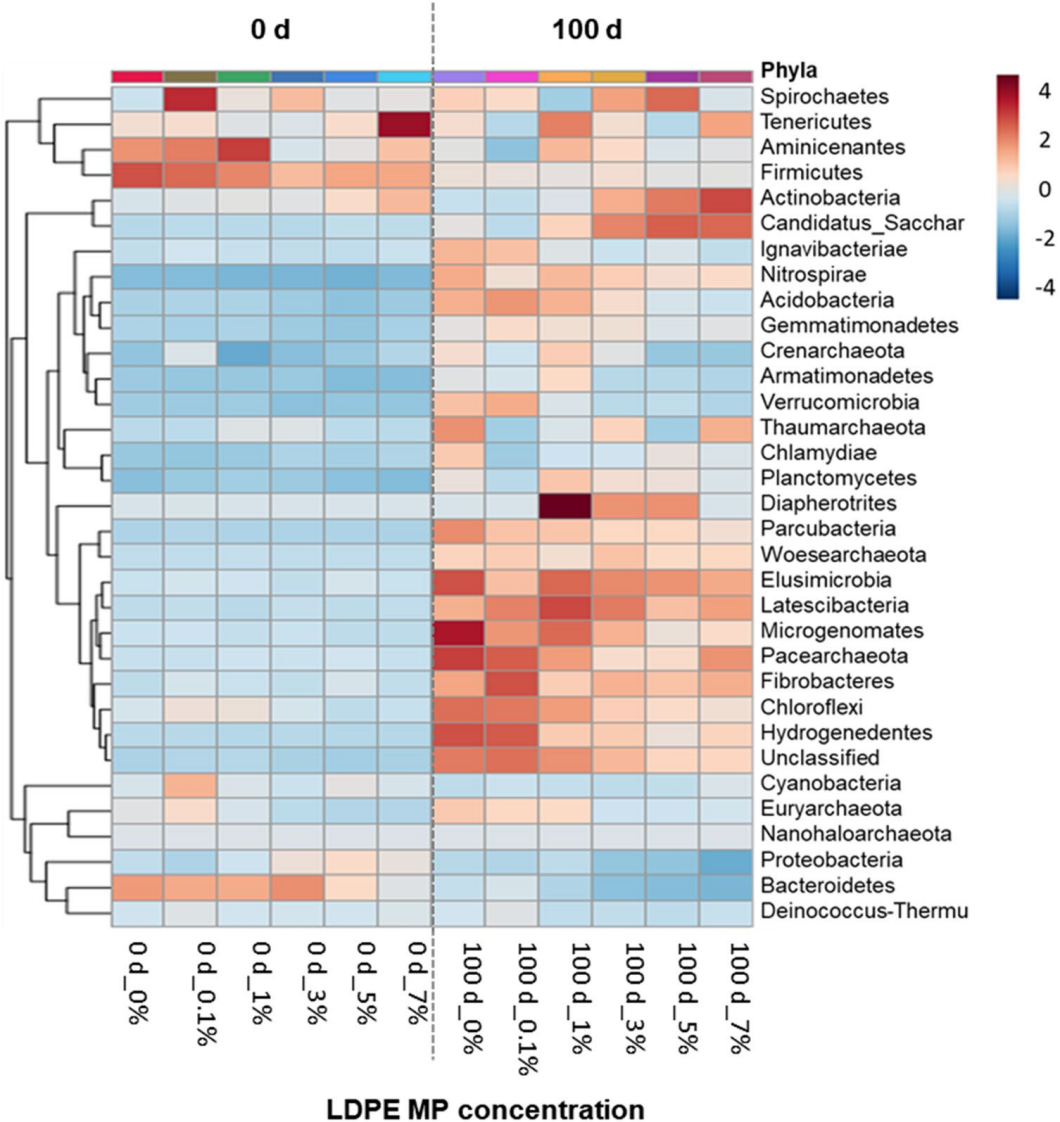
Heat map of bacterial phyla in soil before (0 d) and after (100 d) incubation with varied LDPE MP concentrations at 0, 0.1, 1, 3, 5, and 7%.

A heat map of the bacterial community in terms of phyla in the agricultural soil before incubation (0 d) and after incubation (100 d) according to the LDPE MP concentration is shown in Fig. 4. Overall, the relative abundance of the bacterial community after 100 d of incubation was higher than that before incubation (0 d). Aminicenantes, Firmicutes, and Bacteroidetes showed the highest relative abundance before incubation (0 d); however, the relative abundances of these phyla tended to decrease after incubation (100 d). Elusimicrobia, Latescibacteria, Microgenomates, Pacearchaeota, Fibrobacteres, Chloroflexi, and Hydrogenedentes showed the highest relative abundances at concentrations of 0%, 0.1%, and 1%, after 100 d of incubation. Actinobacteria and Candidatus Saccharibacteria had the highest relative abundances at concentrations of 3, 5, and 7% after incubation for 100 d. These results showed that the prolonged incubation period and the addition of LDPE MP altered the bacterial community structure in the soil. The effects of MPs on soil strongly depend on the exposure time^69^. Similarly, Wang et al.^25^ found that the addition of MP significantly shifted the soil bacterial community structure, and community structure differences increased over the incubation period.

After 100 d of incubation, the relative abundances of Actinobacteria and Candidatus Saccharibacteria tended to increase. In contrast, the relative abundances of Acidobacteria, Microgenomates, Pacearchaeota, Chloroflexi, Hydrogenedentes, and Euryarchaeota tended to decrease with increasing LDPE MP concentrations (Fig. 4). These results reveal that LDPE MP addition stimulated soil microbial abundance, and specific bacterial phyla were present in higher relative abundances across different treatments, indicating that LDPE MPs akin to anthropogenic activity can impose selective pressure on distinct microbial taxa. The effects of MPs on the soil mainly depend on their quantity^3^. MPs can distinctly affect soil properties and exert certain selection pressures on soil microorganisms, thereby altering the community structure and diversity^12^. An increase in the quantity of LDPE MPs in the soil disrupts soil structure, alters porosity, and impairs aeration and water retention^70^. Moreover, increased LDPE MP concentration can affect microbial species, altering soil physical properties, such as soil structure and porosity^15, 71^. Increased soil porosity may enhance airflow in the soil, thereby promoting the abundance of aerobic microorganisms^72^. In contrast, MP exposure substantially decreases the relative abundance of Acidobacteria in fertilized red soil^45^, which could negatively affect soil ecosystems because Acidobacteria plays a key role in soil ecological function^73, 74^. Furthermore, in the 1% LDPE MP treatment, the relative abundance of Diapherotrites after the incubation period was noticeably higher than that of the other phyla (Fig. 4). Their abundance decreased with increasing LDPE MP concentration from 1 to 7%. This implies that 1% LDPE MP is the ideal quantity for the growth and proliferation of some bacterial species belonging to the Diapherotrites. This could be attributed to the formation of a suitable microhabitat for some bacteria at the MP level^26^. Notably, Ignavibacteriae, Acidobacteria, Crenarchaeota, Verrucomicrobia, and Euryarchaeota were more abundant at 0% and 0.1% LDPE MP; however, their abundance decreased with increasing LDPE MP concentrations (Fig. 4), suggesting their low tolerance to high amounts of LDPE MP. Overall, the bacterial heat map demonstrated that the addition of LDPE MPs to the soil tended to increase the bacterial community structure over time, but the abundance of many phyla decreased with increasing LDEP MP concentration. In general, the influence of different quantities of LDPE MP on the soil bacterial communities remains unclear. Thus, further studies are required to elucidate the relationship between MP and microorganisms, which may be highly related to soil properties and MP type, shape, and quantity.

### The relationship of LDPE MP concentration and soil chemical properties with bacterial communities

**Figure 5:**
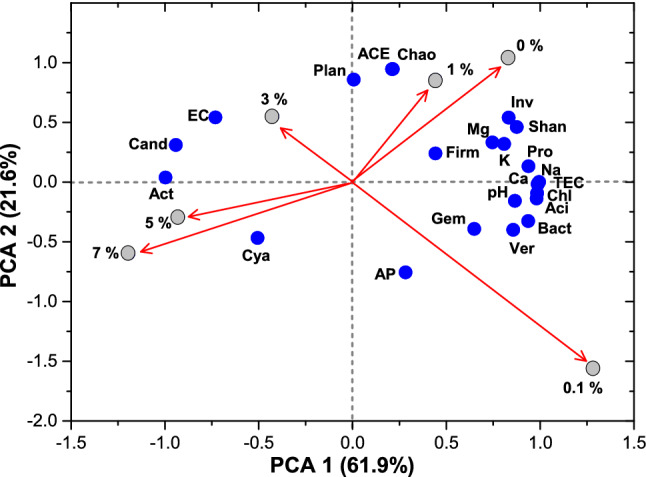
Principal component analysis (PCA) of soil chemical properties (pH, available phosphorous (AP), electrical conductivity (EC), and total exchangeable cations (TEC), total exchangeable Na^+^ (Na), exchangeable K^+^ (K), exchangeable Mg^2+^ (Mg), and exchangeable Ca^2+^ (Ca)), bacterial richness and diversity indices (Chao 1 (Chao), ACE, Shannon (Shan), and InvSimpson (Inv)), and bacterial relative abundance (Acidobacteria (Aci), Actinobacteria (Act), Bacteroidetes (Bact), Candidatus Saccharibacteria (Cand), Chloroflexi (Chl), Cyanobacteria (Cya), Firmicutes (Firm), Gemmatimonadetes (Gem), Planctomycetes (Plan), Proteobacteria (Pro), and Verrucomicrobia (Ver)) with varied LDPE MP concentrations at 0, 0.1, 1, 3, 5, and 7% after 100 d of incubation.

PCA was employed to visualize the correlations between the bacterial community structure and chemical properties of soil with different concentrations of LDPE MPs (Fig. 5). The length and direction of the arrow represent the degree of influence of LDPE MP concentrations on soil chemical and microbial properties and the positive and negative correlations between the two. Components 1 and 2 explained 61.9 and 21.6% of the variance, respectively, accounting for 83.5% of the total variance of the 29 variables. Soil properties, including pH and total exchangeable cations (Na^+^, K^+^, Mg^2+^, and Ca^2+^), were positively correlated with 0% and lower LDPE MP levels (0.1% and 1%) and negatively correlated with higher LDPE MP concentrations (5% and 7%) (Fig. 5). Bacterial richness and diversity indices were positively correlated with the 0% and 1% LDPE MP treatments. These results confirmed that the presence of LDPE MPs in the soil at relatively low quantities (0.1% and 1%) did not have an impact on chemical properties, bacterial richness, and diversity; however, increasing LDPE MPs could alter soil properties and microbial activity. Higher LDPE MP concentrations (3%, 5%, and 7%) were positively correlated with soil EC, indicating that relatively higher MP amounts could increase soil EC.

Acidobacteria, Bacteroidetes, Chloroflexi, Firmicutes, Gemmatimonadetes, Proteobacteria, and Verrucomicrobia were positively correlated with LDPE MP concentrations of 0%, 0.1%, and 1%. Furthermore, these phyla were negatively correlated with LDPE MP concentrations of 5% and 7%. These findings demonstrate that bacterial species belonging to the aforementioned phyla are vulnerable to higher quantities of LDPE MPs. Thus, soil contamination with high amounts of plastics can threaten bacterial abundance and activity in the soil. In contrast, Actinobacteria, Candidatus Saccharibacteria, and Cyanobacteria showed strong positive correlations with the LDPE MP concentrations of 5% and 7%. This implies that bacteria belonging to the aforementioned phyla can grow well under high LDPE MP pollution owing to their LDPE MP tolerance. Similarly, Ren et al.^75^ reported a reduced abundance of Acidobacteria, Bacteroidetes, Gemmatimonadetes, and Proteobacteria and an increased abundance of Actinobacteria in MP-contaminated soil. Acidobacteria, Bacteroidetes, Chloroflexi, Firmicutes, Gemmatimonadetes, Proteobacteria, and Verrucomicrobia were positively correlated with soil pH and exchangeable cation content (Na^+^, K^+^, Mg^2+^, and Ca^2+^). Actinobacteria, Candidatus Saccharibacteria, and Cyanobacteria were positively correlated with the soil EC. These findings further confirmed that changes in soil chemical properties caused by LDPE MPs lead to a greater abundance of bacterial phyla. Changes induced by MPs in the soil may exert a favorable or unfavorable impact on certain soil microorganisms, thereby affecting the soil microbial community structure^76^. Our results showed that LDPE MP addition promoted the growth of tolerant bacteria but impeded that of sensitive bacteria in the soil.

## Conclusion

In summary, the addition of LDPE MPs to soil increased the soil EC at LDPE MP concentrations ranging from 1 to 7%. The total exchangeable cation content decreased at higher LDPE MP concentrations (3–7%), whereas soil pH was not significantly affected by LDPE MP addition. Incubation time increased the overall soil microbial properties in terms of richness, diversity, and relative abundance of the bacterial community. However, at the end of the incubation period, the Chao1 and Ace indices decreased with the addition of 0.1% LDPE MP. Shannon and Invsimpson indices were relatively higher at 0% and 1% LDPE MP addition when compared with those of the other treatments. The relative abundance of Proteobacteria, Actinobacteria, and Acidobacteria was higher in all treatments. The abundance of Actinobacteria increased with an increase in the LDPE MP concentrations and showed a positive correlation with higher LDPE MP concentrations (5% and 7%). Moreover, PCA showed that the presence of LDPE MPs in relatively low quantities in the soil did not affect the chemical properties and bacterial richness, diversity, and abundance. However, increasing the quantity of LDPE MPs may affect soil properties and microbial properties. These results provide new insights into the impact of LDPE MPs on agricultural soil and highlight the important role of LDPE in the soil. Further research is required to determine the impact of various types of MPs on microorganisms, macro-organisms, and higher plants in various agroecosystems. Moreover, field experiments must be conducted in various crop systems under different climatic conditions involving diverse soil types, which will be advantageous for evaluating the ecological impact of plastic mulch pollution on agricultural soils, ecosystems, and ecosystem functions.

### Supplementary Information

Supplementary Tables.

## Data Availability

The data available upon a reasonable request to the corresponding author.
