# Supplementary material for: Low-density polyethylene microplastics alter chemical properties and microbial communities in agricultural soil
Source: Sci Rep. 2023 Sep 28;13:16276. doi: 10.1038/s41598-023-42285-w (PMC10539289; doi:10.1038/s41598-023-42285-w)
Supplement: Supplementary file 1 — Supplementary Tables. [file 41598_2023_42285_MOESM1_ESM.docx]

**Supporting Information**

**Low-density Polyethylene (LDPE) Microplastics Alter Chemical Properties and Microbial** **Communities in Agricultural Soil**

Kumuduni Niroshika Palansooriya^1,2,3^, Mee Kyung Sang^4^, Ali El-Naggar^5,2^, Liang Shi^6^, Scott X. Chang^2^, Jwakyung Sung^7^, Wei Zhang^1,8,**^,Yong Sik Ok^1,9,*^

^1^ Korea Biochar Research Center, APRU Sustainable Waste Management Program & Division of Environmental Science and Ecological Engineering, Korea University, Seoul 02841, Republic of Korea

^2^ Department of Renewable Resources, University of Alberta, Edmonton, Alberta Canada T6G 2E3

^3^State Key Laboratory of Subtropical Silviculture, Zhejiang A&F University, Hangzhou 311300, China

^4^ Division of Agricultural Microbiology, National Institute of Agricultural Science, Rural Development Administration, Wanju 55365, Republic of Korea

^5^ Department of Soil Sciences, Faculty of Agriculture, Ain Shams University, Cairo 11241, Egypt

^6^ College of Life Sciences, Nanjing Agricultural University, Nanjing 210095, China

^7^ Department of Crop Science, College of Agriculture, Life Science and Environmental Chemistry, Chungbuk National University, Cheongju, Chungcheongbuk-do 28644, Korea

^8^ Institute of Environmental Research at Greater Bay Area; Key Laboratory for Water Quality and Conservation of the Pearl River Delta, Ministry of Education, Guangzhou University, Guangzhou, 510006, China

^9^ Institute of Green Manufacturing Technology, College of Engineering, Korea University, Seoul 02841, Korea

*Corresponding author:

Yong Sik Ok; E-mail: [yongsikok@korea.ac.kr](mailto:yongsikok@korea.ac.kr); Tel: +82-2-3290-3044

** Co-corresponding author:

Wei Zhang; E-mail: [zh_wei@gzhu.edu.cn](mailto:zh_wei@gzhu.edu.cn); Tel: +86-20-3741-2113

**Table S1**. Bacterial richness and diversity after incubation based on bacterial diversity indices Chao1, Ace, Shannon, and Invsimpson as affected by low-density polyethylene (LDPE) microplastic (MP) concentrations.

| LDPE MP concentration | 100 days | | | |
| --- | --- | --- | --- | --- |
|  | Chao1 | Ace | Shannon | Invsimpson |
| 0 % | 4774±120 a | 4866±147 a | 6.84±0.027 a | 302±10.8 a |
| 0.1 % | 4338±58 b | 4397±40 b | 6.68±0.010 ab | 206±1.0 bc |
| 1 % | 4721±260 a | 4796±315 ab | 6.83±0.111 a | 301±56.3 a |
| 3 % | 4674±121 ab | 4776±123 ab | 6.71±0.081 ab | 229±39.8 ab |
| 5 % | 4479±207 ab | 4543±225 ab | 6.54±0.107 b | 157±28.6 bc |
| 7 % | 4551±172 ab | 4623±147 ab | 6.48±0.189 b | 133±52.3 c |

Values are given as the average of four replicates, followed by ± standard deviation. Different letters next to the standard deviation indicate significant differences among the LDPE MP concentrations at *p* < 0.05 (least significant difference test) within each index.

**Table S2**. Relative abundance (%) of bacterial community in agricultural soil after incubation as affected by low-density polyethylene (LDPE) microplastic (MP) concentrations.

|  | LDPE MP concentration | | | | | |
| --- | --- | --- | --- | --- | --- | --- |
| **Phyla** | **0%** | **0.1%** | **1%** | **3%** | **5%** | **7%** |
| Acidobacteria | 15.8±0.76 a | 17.2±0.23 a | 15.8±2.1 a | 12.8±1.45 b | 10.5±1.32 c | 9.83±1.20 c |
| Actinobacteria | 7.82±0.39 d | 7.51±0.13 d | 10.6±0.93 d | 21.5±3.48 c | 26.9±2.8 b | 31.8±5.89 a |
| Bacteroidetes | 9.30±0.50 b | 10.7±0.09 a | 7.89±0.54 c | 5.33±0.67 d | 5.14±0.88 de | 4.40±0.44 e |
| Candidatus_Saccharibacteria | 1.54±0.83 bc | 0.68±0.01 c | 2.39±0.91 b | 3.91±0.10 a | 4.52±0.61 a | 4.42±0.80 a |
| Chloroflexi | 10.0±0.69 a | 9.82±0.64 a | 8.92±0.97 a | 7.38±1.22 b | 6.88±0.64 b | 6.48±0.85 b |
| Cyanobacteria | 0.44±0.09 b | 0.63±0.01 ab | 0.53±0.15 b | 0.42±0.07 b | 0.51±0.09 b | 0.95±0.59 a |
| Firmicutes | 5.21±0.91 ab | 4.96±0.07 ab | 4.57±1.03 ab | 5.73±1.46 a | 4.35±0.44 b | 4.31±0.36 b |
| Gemmatimonadetes | 1.95±0.08 bc | 2.25±0.01 a | 2.12±0.09 ab | 2.09±0.29 ab | 1.83±0.18 c | 1.91±0.04 bc |
| Planctomycetes | 2.49±0.19 b | 1.69±0.03 c | 3.22±0.86 a | 2.66±0.12 b | 2.54±0.35 b | 2.19±0.36 bc |
| Proteobacteria | 32.4±2.37 a | 31.8±0.22 ab | 33.0±3.85 a | 29.1±1.24 bc | 28.8±1.36 c | 25.8±1.64 d |
| Verrucomicrobia | 2.58±0.39 a | 2.82±0.05 a | 1.81±0.17 b | 1.47±0.23 c | 1.54±0.18 bc | 1.38±0.23 c |
| Others | 3.09±0.16 a | 2.45±0.03 b | 2.46±0.30 b | 1.94±0.35 c | 1.71±0.29 c | 1.69±0.27 c |
| Unclassified | 7.26±0.25 ab | 7.45±0.12 a | 6.75±0.32 b | 5.72±0.87 c | 4.87±0.37 d | 4.84±0.43 d |

Values are given as the average of four replicates, followed by ± standard deviation. Different letters next to the standard deviation indicate significant differences among the LDPE MP concentrations at *p* < 0.05 (least significant difference test) within each phylum.
